# A Novel Computerized Cognitive Test for the Detection of Mild Cognitive Impairment and Its Association with Neurodegeneration in Alzheimer’s Disease Prone Brain Regions

**DOI:** 10.4236/aad.2023.123004

**Published:** 2023-09-12

**Authors:** Rosie E. Curiel Cid, D. Diane Zheng, Marcela Kitaigorodsky, Malek Adjouadi, Elizabeth A. Crocco, Mike Georgiou, Christian Gonzalez-Jimenez, Alexandra Ortega, Mohammed Goryawala, Natalya Nagornaya, Pradip Pattany, Efrosyni Sfakianaki, Ubbo Visser, David A. Loewenstein

**Affiliations:** 1 Center for Cognitive Neuroscience and Aging and Department of Psychiatry and Behavioral Sciences, Miller School of Medicine, University of Miami, Miami, Florida, USA; 2 Center for Advanced Technology and Education, Department of Electrical and Computer Engineering, College of Engineering and Computing, Florida International University, Miami, Florida, USA; 3 Department of Radiology and Nuclear Medicine, Miller School of Medicine, University of Miami, Miami, Florida, USA; 4 Department of Computer Science, University of Miami, Miami, Florida, USA

**Keywords:** Mild Cognitive Impairment, Proactive Semantic Interference, MRI Volume, Cortical Thickness, LASSI-L

## Abstract

During the prodromal stage of Alzheimer’s disease (AD), neurodegenerative changes can be identified by measuring volumetric loss in AD-prone brain regions on MRI. Cognitive assessments that are sensitive enough to measure the early brain-behavior manifestations of AD and that correlate with biomarkers of neurodegeneration are needed to identify and monitor individuals at risk for dementia. Weak sensitivity to early cognitive change has been a major limitation of traditional cognitive assessments. In this study, we focused on expanding our previous work by determining whether a digitized cognitive stress test, the Loewenstein-Acevedo Scales for Semantic Interference and Learning, Brief Computerized Version (LASSI-BC) could differentiate between Cognitively Unimpaired (CU) and amnestic Mild Cognitive Impairment (aMCI) groups. A second focus was to correlate LASSI-BC performance to volumetric reductions in AD-prone brain regions. Data was gathered from 111 older adults who were comprehensively evaluated and administered the LASSI-BC. Eighty-seven of these participants (51 CU; 36 aMCI) underwent MR imaging. The volumes of 12 AD-prone brain regions were related to LASSI-BC and other memory tests correcting for False Discovery Rate (FDR). Results indicated that, even after adjusting for initial learning ability, the failure to recover from proactive semantic interference (frPSI) on the LASSI-BC differentiated between CU and aMCI groups. An optimal combination of frPSI and initial learning strength on the LASSI-BC yielded an area under the ROC curve of 0.876 (76.1% sensitivity, 82.7% specificity). Further, frPSI on the LASSI-BC was associated with volumetric reductions in the hippocampus, amygdala, inferior temporal lobes, precuneus, and posterior cingulate.

## 1. Introduction

There have been remarkable advances related to the identification of biological markers associated with preclinical Alzheimer’s disease (AD) and Alzheimer’s disease related dementias (ADRD) using both neuroimaging and fluid-based markers of AD pathology [1] [2]. Research suggests that during the prodromal stage of AD, also known as Mild Cognitive Impairment (MCI), neurodegenerative changes can be identified by way of measuring volumetric loss in AD-prone brain regions on MRI [3] [4]. Despite continuous advances to identify and refine biomarkers of AD, and the growing consensus that traditional cognitive assessment paradigms are insensitive to early and subtle cognitive loss [5] [6] [7], the way the field measures early cognitive decline during pre-dementia stages of AD, remains mostly unchanged [8]. Although conventional cognitive assessments have historically proven useful for clinical practice and have aided in longitudinal research studies, the extent of their utility has been questioned, particularly as the field advances in its efforts to measure pre-clinical manifestations of AD that correlate with biomarkers of amyloid, tau and/or neurodegeneration [9]. Identifying subtle cognitive loss is of paramount importance for dementia prevention, as early interventions are likely to delay the clinical onset of impending disease [5] [6] [10].

The Loewenstein-Acevedo Scale of Semantic Interference and Learning (LASSI-L) has shown great utility in detecting cognitive changes during the preclinical stages of AD [11] [12] and has outperformed other widely used memory measures in detecting prodromal states in both English and Spanish [13]. In this cognitive stress paradigm, the LASSI-L employs controlled learning and cued recall to maximize the storage of 15 words (List A) belonging to three semantic categories (fruits, musical instruments, and articles of clothing). This is followed by the administration of different targets representing these same semantic categories that serve to elicit proactive semantic interference (PSI: old learning interfering with new learning). Unlike other traditional paradigms, the LASSI-L then facilitates the assessment of an individual’s ability to recover from PSI through an additional learning trial of the competing list. A growing body of evidence indicates that maximum learning of the initial targets, PSI, frPSI and semantic intrusion errors on the LASSI-L are very sensitive in discriminating between older adults who are cognitively healthy and those with PreMCI or MCI due to AD with amyloid PET biomarker positivity [12] [14] [15] [16]. Multiple studies highlighted that LASSI-L deficits, particularly frPSI, are related to volumetric reductions in AD-prone brain regions [12] [17] [18] [19]. Further, even in cognitively unimpaired older adults with otherwise normal performance on a traditional neuropsychological battery, these AD-salient cognitive deficits were also associated with increased amyloid load in AD-prone areas [11].

Traditional cognitive tests for the assessment and screening of MCI and dementia remain ubiquitously used, although it is generally recognized that most paper-and-pencil tests are lengthy, vulnerable to human error (*i.e.*, administration and scoring), labor-intensive, and prone to practice effects [5]. Moreover, most of these measures have not been subjected to examination for cultural and language biases [20] [21]. To mitigate some of these limitations, some test developers have implemented computer technologies as a suitable alternative; this growing trend of computer-based digital test development offers advantages, such as cost and time savings, greater potential for remotely delivered administration, more uniform and standardized administration procedures, enhanced presentation of stimuli, accurate recording of responses, automated scoring, and real-time data entry. Available systematic reviews have identified more than a dozen computerized measures designed to detect dementia or MCI [22] [23], with most of these tests being adaptations from traditional paradigms; these include the CogState Brief Battery [24] [25], Computer Assessment of Mild Cognitive Impairment (CAMCI) [26], Cambridge Neuropsychological Test Automated Battery (CANTAB) [27], and the Cognition Battery from the National Institutes of Health (NIH) Toolbox [28].

In a recent meta-analysis, Chan and colleagues [29], compared the performance of computerized and paper-and-pencil memory tests among persons diagnosed with MCI and dementia. The authors concluded that the diagnostic performance of some computerized measures was comparable to traditional assessments. While these findings provide evidence that computerized testing paradigms may be a viable alternative to standard modes of psychometric assessment, the psychometric properties of these instruments, such as reliability and validity, have varied, and many have lacked the sensitivity and specificity needed to identify and discriminate early stages of MCI due to AD [22] [29]. Considering the above, computerized measures that use novel cognitive paradigms that are both sensitive and specific to early cognitive changes in AD and converge with biomarkers remain sorely needed.

Given the promising results of the LASSI-L cognitive stress test, CurielCid and colleagues [30] recently developed the LASSI-BC, a brief computerized version of the LASSI-L that incorporates all the well-established measures that have shown discriminative validity (e.g., controlled learning, PSI, frPSI) in the paper-and-pencil LASSI-L. The LASSI-BC does not require a skilled examiner, is web-based, and can remotely run on most browser-capable devices. It is both intuitive and appropriate for use among older adults that are either predominantly English or Spanish-speaking and who have varying ethnic/cultural backgrounds, including Hispanic/Latinos and African Americans [12] [31]. The LASSI-BC has good test-retest reliability for participants diagnosed with aMCI and based on ROC analyses and logistic regression, this version also showed high discriminant validity in differentiating a modest number of aMCI from CU controls [30]. The aims of the current investigation were to expand upon our previous findings using a larger sample to determine the ability of the LASSI-BC to differentiate CU older adults from their aMCI counterparts and to examine whether performance on the LASSI-BC was associated with MRI volumes within brain regions that have shown susceptibility to AD-prone neurodegeneration [32]. We also examined these associations with other commonly used memory measures.

## 2. Methods

### 2.1. Participants

One hundred ten older adults from an NIA-funded R01 study were recruited into this IRB approved investigation at the University of Miami Miller School of Medicine. Participants were evaluated using a standard clinical assessment protocol, which included the Clinical Dementia Rating Scale (CDR) [33], and the Mini-mental State Examination (MMSE) [34]. Experienced clinicians who were blind to the neuropsychological test results and had formal training in administering the CDR and MMSE assessed memory and other clinical and cognitive complaints. To be included in the study, participants must be at least 60 years old, community-dwellers, independent in their activities of daily living, had knowledgeable collateral informants, and did not meet DSM-V criteria for Major Neurocognitive Disorder, an active Mood or Psychotic Disorder, or any other DSM-V Axis I neuropsychiatric disorder [35]. In cases where there was evidence of memory decline by history and/or clinical examination, a Global score of 0.5 was given on the CDR and a probable diagnosis of amnestic MCI (aMCI), was assigned, pending the results of formal neuropsychological testing. Next, a standard neuropsychological battery was uniformly administered across groups independent of the clinical examination and in the participants’ dominant and/or preferred language by experienced bilingual (English/Spanish) psychometrists.

### 2.2. Amnestic MCI Group (aMCI; n = 46)

Based on the independent clinical interview and performance on the neuropsychological tests, an individual was classified as aMCI with a single amnestic deficit, or with an amnestic deficit plus additional non-amnestic deficits if there were: a) subjective memory complaints by the participant and/or collateral informant; b) evidence by clinical evaluation or history of memory and/or other cognitive decline; c) Global Clinical Dementia Rating scale of 0.5; d) one or more memory measures fell below normal limits at 1.5 SD or more relative to age, education, and language-adjusted normative data. The mean age of the aMCI sample was 73.8 (SD = 8.5 years) and the average level of education was 14.3 (SD = 4.3 years). Female participants comprised 58.7% of the aMCI cohort and 50% were evaluated in English, their dominant language. The mean MMSE score was 26.5 (SD = 2.2, range 23 to 30).

### 2.3. Cognitively Unimpaired Group (CU; n = 81)

Participants were classified as CU if all of the following criteria were met: a) no subjective cognitive complaints made by the participant and a collateral informant; b) no evidence by clinical evaluation or history of memory or other cognitive decline after an extensive interview with the participant and an informant; c) Global CDR score of 0.0; d) performance on all traditional neuropsychological tests noted above was not more than 1.0 SD below normal limits for age, education, and language-adjusted normative data. Overall, CU controls were slightly younger 69.8 (SD = 6.1 years) than the aMCI group and slightly more educated than aMCI cohort [16.2 (SD = 2.6 years)]. Female participants comprised the majority of 76.5% of this group and 67% were evaluated in English. The mean MMSE score was 28.9 (SD = 1.2). There were no statistically significant differences in race/ethnicity and language of testing between the two groups (reference Table 1).

### 2.4. Neuropsychological Measures

The Loewenstein-Acevedo Scales for Semantic Interference and Learning, Brief Computerized Version (LASSI-BC) is the digitalized version of the LASSI-L cognitive stress test, a novel cognitive assessment paradigm designed to elicit early AD-related cognitive decline. This computerized measure, which is briefer than the paper-and-pencil LASSI-L, takes approximately 10 to 12 minutes to complete. The LASSI-BC contains the elements of the original LASSI-L which demonstrated the greatest differentiation between aMCI, PreMCI, and CU older adults in multiple previous cross-sectional [11] [30] [36] and longitudinal follow-up studies [14] [16]. The LASSI-BC is a remotely accessible test that can be run on devices that support Google Chrome, including desktop computers, laptops, tablets, or even smartphones. While the LASSI-BC is a fully self-administered test with all verbal responses recorded and scored by the computer, for the purposes of this study, a trained study team member was present for each administration to systematically record responses, which provided a double check on the accuracy of data. The LASSI-BC is available in both English and Spanish. A thorough description of the test and its psychometric properties was written by Curiel Cid and colleagues [30].

Primary LASSI-BC measures used in this study include the second cued recall score for List A (maximum learning), first cued recall score for List B (susceptibility to PSI), and second cued recall score of List B (frPSI). Semantic intrusion errors made on these subscales were also examined given that these have shown to be related to the presence of amyloid pathology in the paper-and-pencil version of test [15].

The remainder of the neuropsychological battery that was used along with the clinical evaluation for classifying participants into diagnostic groups included the Hopkins Verbal Learning Test (HVLT-R) [37], delayed paragraph recall of the National Alzheimer’s Coordinating Center Uniform Data Set (NACC UDS) [38], Controlled Oral Word Association Test: Category Fluency [39], Block Design subtest of the Wechsler Adult Intelligence Scale, Fourth Edition (WAIS-IV) [40], and the Trail Making Test (Parts A and B) [41]. The LASSI-BC was not used for diagnostic determination to avoid any circularity in confounding elements of initial diagnosis with primary outcomes.

### 2.5. MRI Measurements

51 CU and 36 aMCI participants underwent MRI scanning using a GE Discovery MR750 3T (GE, Waukesha, WI, USA) MRI scanner located at the University of Miami School of Medicine. Brain parcellation was obtained using a 3D T1-weighted sequence (MPRAGE) with 1.0 mm isotropic resolution. Free Surfer Version 6.0 software (http://surfer.nmr.mgh.harvard.edu) was employed to assess volumes in Alzheimer’s signature regions specified by Dickerson and colleagues [32] and from our previous work [11] [19] which included regions such as the hippocampus, entorhinal cortex, amygdala, para-hippocampal gyrus, inferior temporal lobule, temporal pole, supramarginal, superior parietal, precuneus, rostral middle frontal and superior frontal areas. All volumes of the brain regions were normalized by dividing by the total intracranial volume.

### 2.6. Statistical Analysis

The distribution of demographic factors and neuropsychological measures were calculated and compared between the two diagnostics groups using χ^2^ test for categorical variables and T-test for continuous variables. Comparative scores for each diagnostic group on the LASSI-L measures were adjusted for statistically significant demographic variables using a one-way analysis of covariance. Binary logistic regression was performed to examine the ability of LASSI-BC measures to differentiate CU vs. aMCI cases. The outcome variable of the logistic regression was the binary cognitive diagnosis (CU vs. aMCI) and the predictors were the LASSI-BC measures (controlling for age, sex, education, testing language, and global cognitive performance as measured by MMSE total score). Odds ratio (OR) and corresponding 95% confidence interval (CI) of aMCI diagnosis was reported with an OR of less than one, indicating less likely to be aMCI. Receiver Operator Characteristic (ROC) curves were calculated for each LASSI-BC measure to determine their ability to classify aMCI cases from their CU counterparts. The area under the ROC curve and 95% confidence interval were reported. The Youden Index, which identifies the optimal cutoff and corresponding sensitivity, and specificity was also reported. A combination of LASSI-BC subscales measuring maximum learning and frPSI and were also examined under the ROC curve.

For the subgroup of 87 older adults who underwent imaging, we examined the association between LASSI-BC and traditional memory and non-memory measures with 13 different AD prone brain regions using structural MRI. Based on our previous work using the paper-and-pencil form of the test, we had an a priori hypothesis that performance on LASSI-BC A2 Cued Recall (maximum learning), B1 Cued Recall and B2 Cued recall would be related to AD sensitive regions such as the hippocampus and precuneus in participants with aMCI. We examined the normality of each variable through normal probability plot and the Shapiro-Wilk normality test. Pearson correlation coefficients within aMCI and CU groups were computed separately, and the correlation coefficient matrices were constructed. To adjust for multiple test comparisons, FDR analysis were performed for each-test-wise contrast to adjust the p-values. Only p-values corrected for FDR using methods by Benjamini and Hochberg [42] that are <0.05 were considered. We further calculated the Pearson correlation coefficients while controlling for maximum learning capacity (Cued A2 recall) to determine whether performance on Cued B1 or Cued B2 had independent explanatory power beyond maximum learning capacity (Cued A2 recall).

## 3. Results

On average, CU participants scored higher on the MMSE (28.9 vs. 26.5, p < 0.001). Unsurprisingly, the CU group also scored higher on the HVLT-R total recall (adjusted mean 24.1 vs. 17.7, p < 0.001) and NACC delayed story passage (adjusted mean 12.4 vs. 7.7, p < 0.001), given that these measures were used to assign participants to diagnostic groups. Importantly, performance on the LASSI-BC measures were not employed in the diagnostic process. Participants with aMCI scored lower on LASSI-BC A2 Cued Recall (maximum learning) (covariate adjusted mean 10.7 vs. 13.1, p < 0.001), B1 Cued Recall and intrusion errors (PSI) (adjusted mean 5.4 vs. 7.5 recall, 4.0 vs. 1.7 intrusion, both p < 0.001) and B2 Cued Recall and intrusion errors (frPSI) (adjusted mean 7.3 vs. 10.9 recall, 3.5 vs. 1.3 intrusion, both p < 0.001). The mean of LASSI-BC Cued B1 and Cued B2 recalls and intrusions after further adjusting for A2Cued recall (maximum learning) were also reported. As indicated in Table 2, the mean values of both Cued B2 recall and intrusions (frPSI) remained statistically different between CU and aMCI after adjusting for maximum learning and the covariates (10.4 vs. 8.8 Cued B2 recall, and 1.5 vs. 3.1 Cued B2 intrusions, both p < 0.01). The mean differences also held for measures of PSI (7.2 vs 6.0, p < 0.01 Cued B1 recall) and intrusion errors made when confronted with PSI (Cued B1 intrusions 2.1 vs. 3.4, p < 0.05).

A logistic regression model controlling for age, sex, education, testing language, and MMSE score showed that LASSI-BC measures were very effective in differentiating CU from aMCI (Table 3). For example, a one-point increase on LASSI-BC Cued A2 recall score (maximum learning) was associated with 48% less likelihood of being diagnosed as aMCI (OR 0.48, 95% CI [0.33, 0.70], p < 0.001). We then further adjusted the regression model by Cued A2 recall demonstrating the discriminating ability of frPSI (Cued B2) in relation to the primary learning effect (Table 3). Cued B2 recall and intrusion errors on this subscale both remained statistically significant in differentiating CU vs. aMCI after adjusting for initial learning (Cued A2 recall). The odds ratios for LASSI-BC Cued B2 recall and intrusions were 0.74 [0.58, 0.94] and 1.51 [1.11, 2.08] respectively, both p < 0.01.

**Figure 1.**
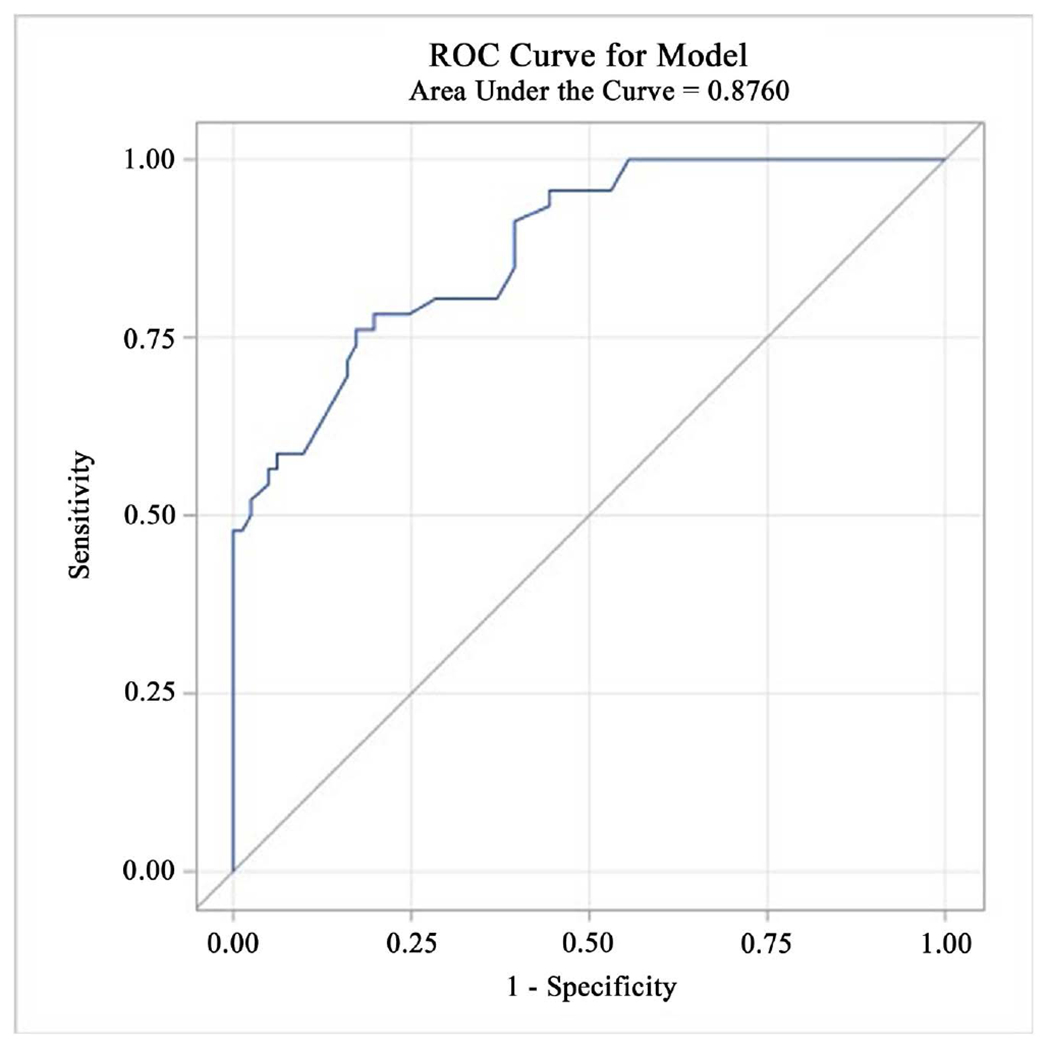
ROC curve of LASSI-BC Cued A2 and Cued B2 Recall in distinguishing between CU vs aMCI groups.

ROC analysis for LASSI-BC A2 Cued Recall (maximum learning) yielded an area under the ROC curved of 0.85% and 95% CI of [0.78 to 0.92] (p < 0.001). A cutoff of >11 by the maximum Youden J index value of 0.56 yielded a sensitivity of 67% and specificity of 89%. For LASSI-BC Cued B2 Recall (frPSI), the area under the ROC curve was 0.82 and 95% CI [0.75, 0.89], p < 0.001. A cutoff of >10 was associated with the maximum Youden J index value of 0.55 and yielded a sensitivity of 87% and specificity of 68%. A combination of these LASSI-BC measures [A2 Cued recall (maximum learning) and Cued B2 recall (frPSI)] yielded an area under ROC curve of 0.876 with 95% CI of [0.82, 0.94] and 76.1% sensitivity and 82.7% specificity (Figure 1).

### LASSI-BC Measures and Regional Brain Volumes on MRI

The associations between LASSI-BC measures and brain volumes of AD prone regions measured by MRI were examined for 51 CU and 36 aMCI participants separately. In this instance, the distribution of age, sex, race, and testing language were similar between groups (all p > 0.05). The aMCI group had less education (14.3 vs. 16.9, p < 0.01) and scored lower on the MMSE (26.6 vs. 29.0, p < 0.01). The aMCI group also scored lower on all LASSI-BC measures, and as expected, HVLT-R total learning and NACC delayed logical memory scores, since these were used for diagnostic classification (all p < 0.01).

Pearson Correlation analyses were conducted separately for each LASSI-BC and standard memory measures. The normality tests indicate the normality assumption was met. To account for multiple comparisons, p-values were adjusted for FDR. Among participants with aMCI, LASSI-BC A2 Cued Recall (maximum learning) was associated with volumes in the hippocampus (r = 0.31; p = 0.049), precuneus (r = 0.52; p = 0.013), inferior temporal lobule (r = 0.41; p= 0.013), superior frontal lobule (r= 0.56; p = 0.003), amygdala (r = 0.43; p = 0.007), posterior cingulate (r = 0.42; p = 0.013), superior parietal lobule (r = 0.41; p = 0.013), rostral middle frontal (r = 0.41, p = 0.013), and supramarginal (r = 0.34, p = 0.039) regions (Table 4).

Among participants with aMCI, after adjusting p-values for FDR, frPSI (LASSI-BC B2 Cued Recall) was associated with volumes of 11 of the 13 brain regions examined: the hippocampus (r = 0.62; p = 0.001), entorhinal cortex (r = 0.33, p = 0.036), precuneus (r= 0.51; p = 0.003), inferior temporal lobule (r = 0.51; p = 0.003), superior frontal lobule (r = 0.49; p = 0.003), amygdala (r = 0.61; p = 0.003), posterior cingulate (r = 0.45; p = 0.007), superior parietal (r = 0.31, p = 0.040), para-hippocampal (r=0.33, p = 0.036), rostral middle frontal (r = 0.35, p = 0.033), and supra marginal (r = 0.37, p = 0.027) regions (Table 4). Performance on HVLT-R immediate recall score, NACC passages immediate and delayed recall were not related to any of the MRI brain volumes measured. The HVLT-R delayed recall score was only associated with the posterior cingulate area (r = 0.45; p = 0.034). Pearson correlation coefficients were calculated while controlling for maximum learning capacity (Cued A2 recall) in the aMCI cohort and adjusted for FDR. Results indicated that LASSI-BC Cued B2 Recall (frPSI) was still highly associated with hippocampal volume (r = 0.57, p = 0.003) and amygdala volume (r = 0.51, p = 0.007). Among the 51 CU cases who underwent MRI, there was no association with neuropsychological measures.

## 4. Discussion

We were able to largely replicate our previous work in an independent sample of older adults diagnosed with aMCI, using the LASSI-BC computerized measure. Results are demonstrative of the fact that maximum learning capacity and frPSI are uniquely and significantly associated with brain volumes in AD prone brain regions among persons with aMCI including the hippocampus, precuneus, inferior temporal lobules, rostral middle frontal areas and temporal pole, among other regions affected early by AD neuropathology in at-risk older adults. Unlike previous studies with LASSI-L [36], we were unable to replicate previous findings of a relationship between frPSI and volumetric reductions in the entorhinal cortex; an interesting finding given the significant correlation between other circuits involving the medial temporal lobe structures. Our present aMCI sample who was administered the LASSI-BC was predominantly community-based, whereas our previous work exploring MRI neurodegeneration with LASSI-L (paper-and-pencil), had a greater admixture of both clinic-based and community-based samples. It is well established that the base rate of underlying AD is higher in those seeking evaluation for memory disorders than in the general community, which may account for the stronger associations between the LASSI-L that was previously studied and volumetric loss in the ERC. Other widely used cognitive tests tapping learning, particularly HVLT-R total recall and NACC delayed passages were not related to brain volumes in the regions studied; however, HVLT delayed recall was associated with the posterior cingulate area in a similar manner. There were no correlations between any neuropsychological measures and neuroimaging among CU.

Among persons with aMCI, measures of association were higher on a measure of maximum learning when compared to frPSI, which raised the question of whether performance deficits captured by frPSI are related to an underlying memory deficit or, whether frPSI independently explained the association. To examine this, we further adjusted the regression model by maximum learning (Cued A2) and showed the independent discriminating ability of frPSI (Cued B2) and semantic intrusion errors that occur on this subscale.

One of the strengths of the current study includes the replication of many of our previous findings, but in a different sample of aMCI participants using similar diagnostic criteria. In addition, we employed methods to control for FDR and to minimize the possibility of family-wise Type 1 errors. Potential weaknesses include a modest number of aMCI participants receiving MRI scans and the inability to discern the performance of larger numbers of diverse ethnic-cultural groups. Those with a diagnosis of aMCI do not necessarily have underlying AD pathology and we plan to obtain as many amyloid PET scans as possible in this growing cohort to examine the LASSI-BC as it relates to specific etiology. As attention is focused on developing tools to detect early cognitive deficits in preclinical stages of neurodegenerative disorders such as AD, it is important to employ paradigms that act as cognitive stress tests to detect subtle deficits among older adults who may have little or no cognitive impairment on traditional neuropsychological measures.

A unique aspect of the LASSI-BC, relative to other computerized cognitive measures, is that it employs a sensitive semantic interference paradigm that has been shown to be a salient early cognitive breakdown in preclinical AD and related to multiple biomarkers. Emerging cognitive tests should also be required to exhibit sensitivity to biomarkers of AD (e.g., amyloid, tau, and neurodegeneration in AD-prone regions). Doing so may address some of the most critical challenges facing clinical trials including proper selection of at-risk participants and monitoring meaningful cognitive change over time. Another added advantage to the LASSI-BC is the ability to undergo this test remotely given that, after the COVID-19 pandemic, telemedicine is on a path of becoming a modal form of healthcare delivery. Kitaigorodsky and colleagues [43] noted that remote care can benefit older adults who lack transportation, are socially isolated, present with physical impediments, live a great distance from a tertiary medical care center, or are vulnerable to contracting infections in person. As such, the development, refinement, and validation and of digital neuropsychological assessments is of paramount importance.

Limitations of the study include a relatively modest number of aMCI cases in relation to CU counterparts, and that these were predominantly female. Further, there was a significant difference in levels of education between cohorts, as on average, aMCI grouping received less schooling. Lastly, the cross-sectional nature of the investigation could also be deemed as a limiting factor. Subsequent studies would be enriched by examining these findings as predictive of longitudinal changes in cognition and including fluid-based markers of neurodegeneration. Subsequent works including diverse ethnic/cultural groups, are required to determine the generalizability of this finding and whether measures susceptible to frPSI are predictive of longitudinal changes in cognition and specific biomarkers [44].

## Figures and Tables

**Table 1.** Comparison between CU and aMCI participants.

	CU	aMCI	p value
N	81	46	
Age (SD)	69.8 (6.1)	73.8 (8.5)	<0.01

Sex			

Female	76.5%	58.7%	0.03
Male	23.5%	41.3%	
Education (range 5 - 21)	16.2 (2.6)	14.3 (4.3)	0.01
Race			
Non-Hispanic White	48.7%	35%	0.18
Hispanic	42.5%	48%	
Other	8.8%	17%	

Language of testing			

English	67%	50%	0.06
Spanish	33%	50%	
MMSE (SD)	28.9 (1.2)	26.5 (2.2)	<0.001
(MMSE Range)	(24 - 30)	(23-30)	

**Covariate-adjusted Means** *			

HVLT-R total	24.1	17.7	<0.001
HVLT-R delayed	7.7	2.3	<0.001
NACC Logical Memory delay	12.4	7.7	<0.001
LASSIBC Cued Recall A2	13.1	10.7	<0.001
LASSIBC Cued Recall B1	7.5	5.4	<0.001
LASSIBC Cued Recall B2	10.9	8.0	<0.001
LASSIBC Cued Intrusion B1	1.7	4.0	<0.001
LASSIBC Cued Intrusion B2	1.3	3.5	<0.001

* Means adjusted for age, sex, and education.

**Table 2.** Mean difference between CU and aMCI adjusting for maximum learning and demographic covariates.

	CU	MCI	p Value
LASSI BC Cued Recall B1	7.2	6.0	0.045
LASSI BC Cued Recall B2	10.4	8.8	<0.01
LASSI BC Intrusions B1	2.1	3.4	<0.01
LASSI BC Intrusions B2	1.5	3.1	<0.01

* Means adjusted for age, sex, education, and LASSI BC Cued recall A2 (maximum learning).

**Table 3.** Logistic regression of LASSI-BC measures in differentiating CU vs. aMCI.

	Odds Ratio of being aMCI	95% Confidence Interval	p value
Adjusted for covariates*			

LASSI BC Cued Recall A2	0.48	[0.33, 0.70]	< 0.001
LASSI BC Cued Recall B1	0.73	[0.59, 0.90]	0.003
LASSI BC Intrusion B1	1.49	[1.17, 1.90]	0.001
LASSI BC Cued Recall B2	0.654	[0.53, 0.81]	< 0.001
LASSI BC Intrusion B2	1.73	[1.29, 2.32]	< 0.001
Adjusted for maximum learning in addition to covariates*			
LASSI BC Cued Recall B1	0.78	[0.62, 0.98]	0.039
LASSI BC Intrusion B1	1.27	[0.98, 1.65]	0.06
LASSI BC Cued Recall B2	0.74	[0.58, 0.94]	<0.01
LASSI BC Intrusion B2	1.51	[1.11, 2.08]	<0.01

* Model controlled for age, sex, education, testing language, and global cognitive functioning.

**Table 4.** The associations between MRI volumes in AD-prone regions and performance on the LASSI-BC and standard memory measures in participants with aMCI.

	LASSI BC Cued Recall A2 (Maximum Learning)	LASSI BC Cued Recall B1 (PSI)	LASSI BC Cued Recall B2 (frPSI)	HVLT-R total	HVLT-R Delay	NACC Delay
Hippocampal volume	**0.31**	0.37	**0.62**	0.01	0.28	−0.08
	(p = 0.049)	(p = 0.065)	(p = 0.001)	(p = 0.560)	(p = 0.083)	(p = 0.758)
ERC volume	0.23	0.28	**0.33**	0.08	0.24	0.09
	(p = 0.111)	(p = 0.072)	(p = 0.036)	(p = 0.422)	(p = 0.118)	(p = 0.758)
Precuneus volume	**0.52**	0.40	**0.51**	0.20	0.22	0.04
	(p = 0.003)	(p = 0.065)	(p = 0.003)	(p = 0.316)	(p = 0.128)	(p = 0.758)
Inferior Temporal volume	**0.41**	0.34	**0.51**	0.15	0.34	0.15
	(p = 0.013)	(p = 0.065)	(p = 0.003)	(p = 0.344)	(p = 0.083)	(p = 0.758)
Superior Frontal volume	0.56	0.29	**0.49**	0.32	0.19	0.16
	(p = 0.003)	(p = 0.072)	(p = 0.003)	(p = 0.262)	(p = 0.161)	(p = 0.758)
Amygdala volume	**0.43**	0.35	**0.61**	0.10	0.29	0.13
	(p = 0.013)	(p = 0.065)	(p = 0.003)	(p = 0.422)	(p = 0.083)	(p = 0.758)
Posterior Cingulate volume	**0.42**	0.33	0.45	0.28	**0.45**	−0.04
	(p = 0.013)	(p = 0.065)	(p = 0.007)	(p = 0.262)	(p = 0.034)	(p = 0.758)
Superior Parietal volume	**0.41**	0.32	**0.31**	0.08	0.32	0.06
	(p = 0.013)	(p = 0.065)	(p = 0.040)	(p = 0.422)	(p = 0.083)	(p = 0.758)
Para-hippocampal	0.22	0.31	**0.33**	0.18	0.30	0.01
	(p = 0.111)	(p = 0.067)	(p = 0.036)	(p = 0.316)	(p = 0.083)	(p = 0.758)
Inferior Lateral Ventricle	−0.16	−0.14	−0.29	−0.06	−0.18	−0.11
	(p = 0.814)	(p = 0.789)	(p = 0.952)	(p = 0.644)	(p = 0.857)	(p = 0.758)
Temporal pole	0.22	−0.10	0.07	0.19	0.29	−0.08
	(p = 0.111)	(p = 0.774)	(p = 0.380)	(p = 0.316)	(p = 0.083)	(p = 0.758)
Rostral Middle Frontal volume	**0.41**	0.12	**0.35**	0.26	−0.03	0.18
	(p = 0.013)	(p = 0.300)	(p = 0.033)	(p = 0.262)	(p = 0.609)	(p = 0.758)
Supra Marginal volume	**0.34**	0.24	**0.37**	−0.03	0.28	−0.12
	(p = 0.039)	(p = 0.106)	(p = 0.027)	(p = 0.617)	(p = 0.083)	(p = 0.758)

p-values are from one-tailed test and FDR adjusted. Bold indicate statistically significant at 0.05 level after False Discovery Rate correction.
